# Connective tissue growth factor promotes cementogenesis and cementum repair via Cx43/β-catenin axis

**DOI:** 10.1186/s13287-022-03149-8

**Published:** 2022-09-06

**Authors:** Zuping Wu, Yuying He, Sirui Chen, Li Zhu, Jiahe Wang, Demao Zhang, Jing Xie, Shujuan Zou, Chenchen Zhou

**Affiliations:** 1 State Key Laboratory of Oral Diseases, West China Hospital of Stomatology, Sichuan University, Chengdu, 610041 China; 2 National Clinical Research Center for Oral Diseases, West China Hospital of Stomatology, Sichuan University, Chengdu, 610041 China; 3 State Key Laboratory of Oral Diseases, West China Hospital of Stomatology, Sichuan University, Chengdu, 610064 Sichuan China

**Keywords:** CTGF, Cx43, Cementum, β-Catenin, Human periodontal stem cells

## Abstract

**Background:**

Orthodontic tooth movement inevitably induces cementum resorption, which is an urgent problem for orthodontists to confront. Human periodontal ligament stem cells (hPDLSCs) exert an important role in the orthodontic tooth movement and exhibit multidirectional differentiation ability in cementum regeneration. Connective tissue growth factor (CTGF) is an important extracellular matrix protein for bone homeostasis and cell differentiation. The purpose of our study was to explore the role of CTGF in cementum repair and cementogenesis and to elucidate its underlying mechanism.

**Methods:**

A cementum defect model was established by tooth movement with heavy forces, and the cementum repair effect of CTGF was observed via micro-CT, HE staining and immunohistochemical staining. RT‒qPCR, western blotting (WB), alizarin red staining and ALP activity experiments verified the mineralization ability of hPDLSCs stimulated with CTGF. The expression of Cx43 in periodontal ligament cells was detected by WB and immunofluorescence (IF) experiments after CTGF stimulation in vivo and in vitro. Subsequently, the mineralization ability of hPDLSCs was observed after application of CTGF and the small interfering RNA Si-Cx43. Additionally, co-intervention via application of the small interfering RNA Si-CTGF and the Cx43 agonist ATRA in hPDLSCs was performed to deepen the mechanistic study. Next, WB, IF experiments and co-immunoprecipitation were conducted to confirm whether CTGF triggers the Cx43/β-catenin axis to regulate cementoblast differentiation of hPDLSCs.

**Results:**

Local oral administration of CTGF to the cementum defects in vivo facilitated cementum repair. CTGF facilitated the cementogenesis of hPDLSCs in a concentration-dependent manner. Cx43 acted as a downstream effector of CTGF to regulate cementoblast differentiation. Si-Cx43 reduced CTGF-induced cementoblast differentiation. The Cx43 agonist ATRA restored the low differentiation capacity induced by Si-CTGF. Further mechanistic studies showed that CTGF triggered the activation of β-catenin in a dose-dependent manner. In addition, co-localization IF analysis and co-immunoprecipitation demonstrated that Cx43 interacted with β-catenin at cell‒cell connections. Si-Cx43 attenuated the substantial expression of β-catenin induced by CTGF. The Cx43 agonist reversed the inhibition of β-catenin induced by Si-CTGF. IF demonstrated that the nuclear importation of β-catenin was related to the immense expression of Cx43 at cell‒cell junctions.

**Conclusions:**

Taken together, these data demonstrate that CTGF promotes cementum repair and cementogenesis through activation of the Cx43/β-catenin signalling axis.

**Supplementary Information:**

The online version contains supplementary material available at 10.1186/s13287-022-03149-8.

## Introduction

Orthodontic-induced external root resorption (OIRR) is an inevitable side effect during orthodontic treatment and is accompanied by cementum resorption [1]. It has been reported that 5% of clients undergo root resorption over 5 mm, which might affect the lifetime of the tooth [1, 2]. Promoting cementum regeneration and root repair has become a clinical issue of great concern to orthodontists. When subjected to excessive compressive forces, odontoclasts become highly activated and initiate the complex process of root resorption [3]. Human periodontal ligament (hPDL) embedded between the alveolar bone and cementum is a significant unmineralized tissue for cementum mineralization and cementum repair [4, 5]. Periodontal ligament stem cells (hPDLSCs) are the basis for maintaining a stable periodontal environment and the long-term stability of teeth and are involved in reversing the pathological process of root resorption caused by trauma and infection [4–6]. Recently, several studies have revealed that growth factors, such as platelet-derived growth factors, promote PDL regeneration, including cementum regeneration [7, 8]. Thus, finding new stimuli to promote cementum repair is critical for both patients and orthodontists.

Cellular communication network factor 2/connective tissue growth factor (CCN2/CTGF) is an extracellular matrix protein that exhibits a cysteine-abundant structure [9, 10]. CTGF exerts diverse effects on cellular activities, such as cell proliferation, migration and differentiation [9–12]. CTGF ablation in mice induces developmental skeletal deformities [12, 13]. Additionally, CTGF serves as potential factor to stimulate the cell multiplication and differentiation of osteoblasts and chondrocytes and enhance the generation of extracellular matrix proteins, such as type I collagen and integrins [10, 14]. During tooth germ development, the expression of CTGF is spatiotemporally restricted and specifically distributed in different odontogenic cells, and inhibiting CTGF expression was found to delay the differentiation of ameloblasts and odontoblasts [11]. Thus, CTGF has been identified as a novel, potent co-stimulator in tooth and bone development. A recent study has found that cementum-related cementoblasts or cementocytes were strongly positive-labelled with CTGF, suggesting that CTGF may play a role in cementum formation [15].

Connexin43 (Cx43) belongs to the gap junction (GJ) protein family [16]. Cx43 in the plasma membrane forms aqueous channels with neighbouring cells to exchange small particles, proteins and second messengers. Thousands of intercellular communication connections between periodontal tissues act as high-speed pathways to exchange biological information. The construction of effective intercellular communication via Cx43 coordinates the function and stability of tissues [16]. Cx43 plays a crucial role in craniofacial development. Oculodentodigital dysplasia (ODDD) results from a point mutation of the Cx43 gene, and is mainly manifested in oral functions, such as the induction of dental caries and accelerated tooth loss [16]. As for periodontal tissue, Cx43 was first identified by Beertsen et al. [17]. During orthodontic tooth movement, the protein abundance of Cx43 in PDL cells at the compressive zone was decreased under hypoxia, and Cx43 expression showed a significant enhancement in the tension zone, suggesting that Cx43 has great importance in bone remodelling [18, 19]. Recently, Cx43 has been identified as a protein marker of cementocytes, indicating that Cx43 may be involved in cementum formation [20]. Cx43 expression directly or indirectly activates multiple signalling pathways. β-catenin, one of the signalling pathways regulated by Cx43, plays a significant role in osteoblasts, periodontal ligament cell differentiation and bone homeostasis [5, 16, 21, 22]. A recent study showed that activation of the β-catenin pathway might contribute to cementum reconstruction. Conditional ablation of β-catenin in Gli1^+^ PDL cells was found to affect cementum development [5]. Cx43 is also vital in bone homeostasis, suggesting that the functions of Cx43 and β-catenin in bone may be linked [4, 21–23]. The regulation of β-catenin by Cx43 is now controversial. A point mutation targeting Cx43 in osteoblasts resulted in Cx43 functional loss, which subsequently inhibited the β-catenin signalling pathway and delayed fracture healing [24, 25]. On the other hand, depletion of Cx43 in osteocytes may activate β-catenin signalling under mechanical loading [26], suggesting that this difference is due to a cell-type dependent mechanism.

A previous study by our team revealed that CTGF promoted cell‒cell communication and cell connection formation in chondrocytes in a Cx43-dependent manner, thus influencing cell biological processes, such as cell migration [27]. β-catenin acts as a signalling pathway downstream of Cx43 to regulate bone homeostasis [28]. Due to the similar biochemical processes shared by cementum regeneration and bone regeneration [20, 29], we hypothesized that CTGF might promote cementum regeneration through the Cx43/β-catenin axis and stimulate cementum repair when OIRR occurs.

## Materials and methods

### Root repair model protocol and local CTGF administration

The establishment of a root repair model was consistent with our previous study [30]. Thirty-six 6-week-old male SD rats weighing approximately 200 ± 10 g were randomly selected for our study. The experimental design was ratified by the Ethics Committee of West China Stomatological Hospital (WCHSIRB-D-2021-601, Additional file 1: Fig. S1). Under anaesthesia, an orthodontic nickel-titanium spring was applied between the left maxillary first molar and incisor with 100 g of force (Additional file 2: Fig. S2A), and then, OIRR was successfully established after two weeks with heavy force loading. After two weeks, the nickel-titanium tension spring was replaced with a ligature wire to maintain the tooth position. Detailed protocols are shown in Additional file 2: Fig. S2B. To calculate the volume of root resorption after 2 weeks, six rats in each group were sacrificed at this timepoint as initial controls (Day 0). Without orthodontic force, the root impaired by heavy orthodontic forces initiates the biological activity of cementum regeneration (Additional file 2: Fig. S2C-E). The rest of the rats were stochastically sorted into the control group and CTGF treatment group. The rats in the CTGF treatment group received 0.5 µg of CTGF, which was injected every two days locally in the buccal gingiva of the left maxillary first molar, while the control group rats only received a 0.1% acetic acid solution injected locally. Rats were sacrificed on the 14th (Day 14) or 28th (Day 28) day (*n* = 6), and the left maxillae were isolated for the following experiments.

### Microcomputed tomography (micro-CT) analysis

The samples were fixed in 4% paraformaldehyde (PFA) for 24 h and then examined using a high-resolution (10 μm) Micro-CT50 system (Scanco Medical, Wangen-Bruttisellen, Switzerland). The detailed analysis of root resorption was consistent with our previous study [30]. The 3D model of the left maxillary first molar was reconstructed using Mimics 21.0 software to observe the absorption craters (Additional file 2: Fig. S2E). The blue-marked area represents the volume of the existing root, while the pink pseudocolour indicates the resorption root area, which was manually delineated in multiple 2D images using Mimics software according to the morphology of the resorption root. The yellow area in Additional file 2: Fig. S2E indicates the area of cementum resorption. For test subjects, we divided the volume of the absorption craters by the root length to obtain resorption volume per millimetre of root length.

### Histological and immunohistochemical (IHC) analysis

After micro-CT, the left maxillary jaws were decalcified with 14% EDTA (pH 7.4) for 1.5 months, dehydrated in ascending graded alcohol, embedded in paraffin, and then cut into 5-μm serial sections. Root resorption morphology was observed by H&E staining (G1340; Solarbio, Beijing, China). Immunohistochemistry was performed after deparaffinization in xylene and then rehydration in descending graded alcohol. Then, the specimens were subjected to heat-induced epitope retrieval. After incubation with 5% BSA, the sections were incubated overnight with anti-Col1 (1:200, 501,352, Zen-Bio, China), anti-Osterix (1:200, Huabio, ER1914-47, China), anti-Runx2 (1:200, 860,139, Zen-Bio, China), anti-CAP (1:200, sc-53947, Santa, America), anti-Cx43 (1:200, 340,279, Zen-Bio, China), and anti-active β-catenin (1:200, 19,807, Cell Signaling Technology, America) antibodies, followed by incubation with the corresponding IgG secondary antibodies. Binding of an avidin–biotin-peroxidase complex to the DAB substrate from a kit (ZLI-9017; Zhongshan BioTech, China) was used to visualize the positive immune signal. ImageJ software was applied to evaluate hPDLSCs with positive expression (Media Cybernetics, Bethesda, MD, USA).

### Isolation, culture, and identification of hPDLSCs

The detailed cell isolation protocol has been described in a previous study [31]. After obtaining informed consent from the subjects, the periodontal ligament tissues of the premolars or third molars were collected from six patients. We obtained ethics approval and consent from the Ethics Committee of West China Stomatological Hospital (WCHSIRB-D-2021-537, Additional file 3: Fig. S3). Detailed demographic information of the enrolled subjects included the following: (1) 12–20 years old, (2) a need to extract premolars due to orthodontic treatment, and (3) those who had any other general diseases or whose teeth had periodontal pockets deeper than 3 mm were excluded from this study. hPDLSCs were cultured as previously described, and periodontal ligament cells were used for subsequent experiments at passages 3–5 [31]. Alkaline phosphatase, alizarin red and crystal violet staining were used to detect multidirectional differentiation ability. Flow cytometry was performed using primary antibodies against STRO1, CD146, CD34 and CD45 (Proteintech Group, USA) on a BD Accuri™ Cflow cytometer (BD Biosciences, Milan, Italy).

### RNA interference for Cx43 and CTGF knockdown

The RNA interference protocol was consistent with a previous study [27]. hPDLSCs were seeded in 24-well, 48-well and 6-well culture dishes; transfected with the corresponding siRNA (Hanbio, China); and transiently transfected using Lipofectamine RNAiMAX (Invitrogen, Burlington, ON, Canada). The specific siRNA sequences are shown below: Si-Cx43 (forward 5′- GCGACAGAAACAAUUCUUC-3′ and reverse 5′-GAAGAAUUGUUUCUGUCGC-3′); Si-CTGF (forward 5′-GCACCAGUGUGAAGACAUATT -3′ and reverse 5′-UAUGUCUUCACACUGGUGCTT -3′). Western blotting was performed to confirm transfection efficiency.

### Alkaline phosphatase (ALP) staining

hPDLSCs were seeded in 48-well culture dishes and cultivated for 7 days in mineralization medium (ascorbic acid (0.05 M), dexamethasone (100 nM), β-glycerophosphate (10 mM)) with CTGF at ascending concentrations (0, 10, 20, 50 ng/ml). After fixation with 4% PFA, the hPDLSCs were examined using a BCIP/NBT detection kit (C3206, Beyotime, China). Briefly, the hPDLSCs were fully stained for 15 min, rinsed with water and stored for observation under a light microscope. To verify whether Cx43 is involved in CTGF-mediated cementogenic differentiation, we concurrently applied both CTGF and Si-Cx43 to hPDLSCs and fixed the cells for ALP staining seven days later. hPDLSCs were also treated with the Cx43 agonist all-trans retinoic acid (ATRA) (R2625, Sigma, America) and Si-CTGF and again fixed for ALP staining seven days later. In addition, the β-catenin agonist LiCl (ST498, Beyotime, China) was applied to verify the role of the β-catenin pathway in CTGF-induced cementoblast differentiation.

### Alizarin red S (ARS) staining

The detailed hPDLSCs treatment protocols were in accordance with the ALP staining experiments described above. hPDLSCs were seeded in 24-well culture dishes and then cultured in mineralization medium for 14 days under a treatment protocol similar to that described above. The hPDLSCs were dyed with 1% ARS (G1452, Solarbio, China) for 30 min to evaluate the degree of mineralization and then bathed with double distilled water for 3 min. Images were observed under a stereoscopic imaging microscope.

### Western blotting (WB)

The detailed hPDLSCs treatment protocols were in accordance with the ALP staining experiments described above. Total protein was extracted with protein lysis buffer (BL504A, Biosharp, China) containing phosphatase inhibitors following the manufacturer's instructions. After denaturation, the proteins were separated via 10% SDS–polyacrylamide gel electrophoresis and transferred to PVDF membranes. After blocking with 5% skim milk for 1 h, the membranes were incubated with the corresponding primary antibody (1:1000) overnight: rabbit anti-Col1; rabbit anti-Osterix; rabbit anti-Runx2; mouse anti-Cap, rabbit anti-Cx43; rabbit anti-CTGF (R24001, Zen-Bio, China); mouse anti-β-catenin (ab237983, Abcam, UK); and rabbit anti-active-β-catenin, followed by incubation with the corresponding secondary antibody and imaging preparation with a Western Blotting Reagent Kit (P90719, Millipore). ImageJ software was applied to evaluate the intensity of the protein bands.

### Quantitative RT-PCR

The detailed hPDLSCs treatment protocols were in accordance with the ALP staining experiments described above. RNA was collected in accordance with the instructions of an RNA Isolation Kit (Bioteke, Beijing, China). Then, 1 µg of RNA was transformed into cDNA with reverse transcriptase (Thermo Fisher Scientific, MA, USA) and subjected to quantitative RT‒PCR (Bio-Rad, CA, USA). The relative expression of targeted genes (Additional file 6: Table S1) was calculated by applying the ΔΔCt method as previously described [31].

### Immunofluorescence and confocal laser scanning microscopy (CLSM)

The detailed immunofluorescence procedure was described previously [27]. The hPDLSCs were fixed in 4% PFA for 15 min. Subsequently, hPDLSCs or tissue sections were permeated with 0.25% Triton X-100 for 10 min. After being sealed with 5% BSA for 1 h, the hPDLSCs or tissue sections were incubated with anti-Cx43 and anti-β-catenin antibodies overnight at 4 °C, followed by incubation with anti-rabbit antibody conjugated with Alexa Fluor 647 (ab150079, Abcam, UK) or anti-mouse antibody conjugated with Alexa Fluor 488 (ab150113, Abcam, UK) at room temperature for 2 h. DAPI (D9542, Sigma-Aldrich, USA) staining was performed to visualize periodontal ligament cell nuclei. The cytoskeleton was dyed with phalloidin (6 μmol/L, Invitrogen, CA). Fluorescence staining images were obtained via CLSM.

### Co-immunoprecipitation (Co-IP)

The detailed procedure was performed according to the manufacturer's instructions (YJ201, Epizyme Biomedical Technology, China). After ice-cold protein lysis of hPDLSCs for 10 min, the samples were incubated overnight with 2.5 µg of rabbit antibody against β-catenin (R22820, Zen-Bio, China). Then, the antibody-protein complexes were mixed with Protein A/G Plus magnetic beads overnight at 4 °C. After immunoprecipitation, the immune complexes were resuspended in SDS-loading buffer after centrifugation and denatured as mentioned above for western blotting experiments.

### Statistical analysis

The data are representative of three independent experiments and are presented as the mean ± standard deviation (SD). One-way ANOVA and Student's t test were conducted using GraphPad Prism version 8 software (GraphPad, San Diego, CA, United States) to calculate the significance of differences between groups.

## Results

### CTGF promoted cementum regeneration after root resorption induced by heavy orthodontic forces

**Fig. 1:**
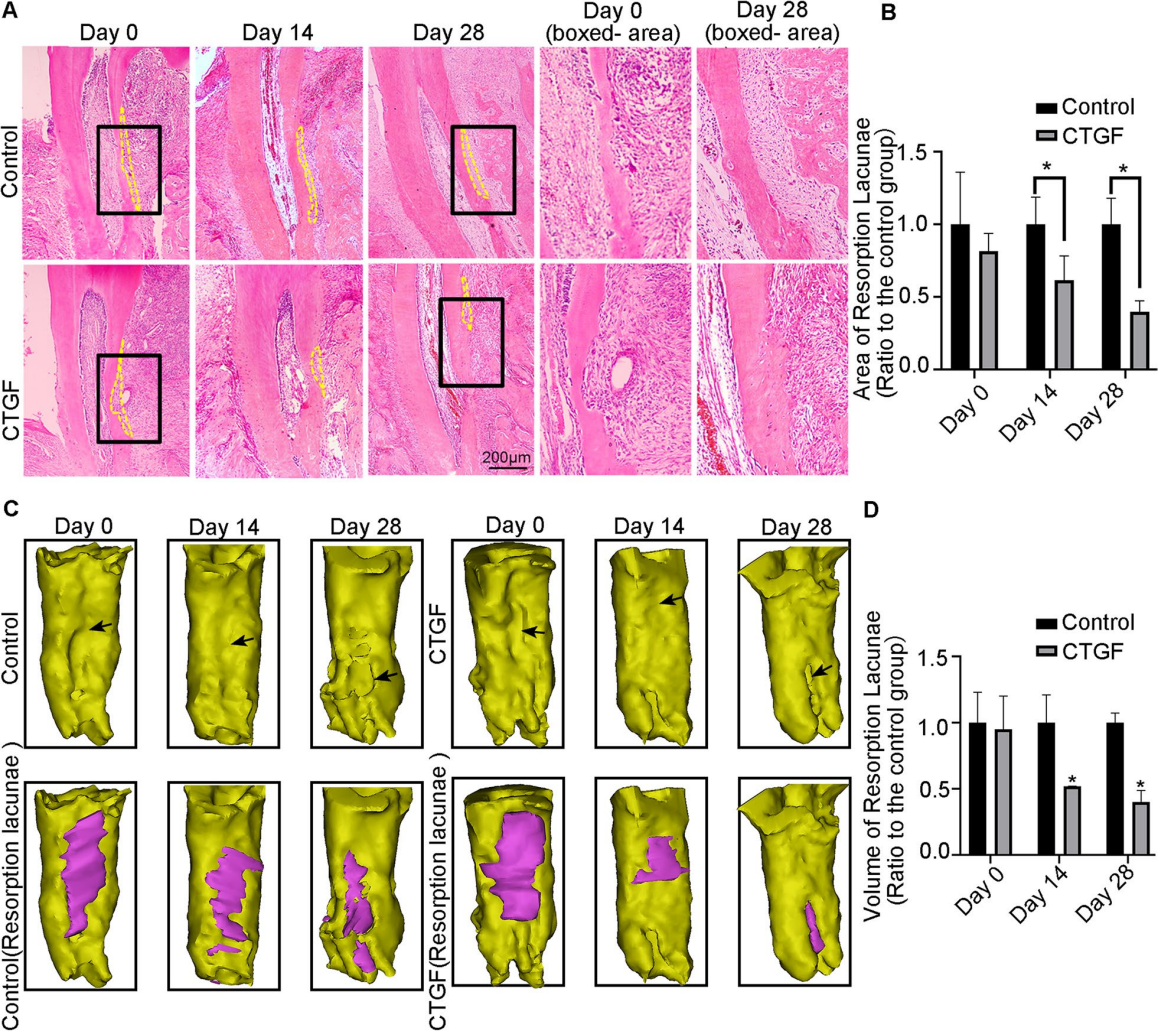
CTGF promoted the repair of root resorption in vivo. **A** Representative images of HE showed the morphology of distal buccal roots of maxillary first molars. **B** Quantification of the area of the resorption lacunae labelled in **A** by yellow dotted line in CTGF-treated rats relative to the untreated rats. Statistical significance is indicated. *p < 0.05. **C** Construction the 3D model of the buccal distal root in the left maxillary first molar by mimics 21. The fuchsia area was reconstructed by the method mentioned in Additional file 2: Fig. S2E. The fuchsia area indicated the root resorption area. The resorption lacunae of the compressive root in the CTGF-stimulated rats were smaller than those of the untreated rats. Black arrows pointed to resorption craters. **D** Quantification indicated the smaller resorption lacunae in CTGF-treated rats relative to the untreated rats. Statistical significance is indicated. *p < 0.05

We successfully established a root resorption model accompanied by root defects and induced relatively obvious tooth movement (Additional file 2: Fig. S2C-D). We reconstructed the buccal mesial root of the first molar following the method shown in Additional file 2: Fig. S1E. We found that heavy orthodontic forces induced tooth root defects. HE staining results demonstrated that the stress might mainly be concentrated in the middle area of roots after heavy orthodontic force loading, where root resorption frequently occurs. With CTGF treatment, the resorption craters on the root surface were gradually filled with regenerated cementum (Fig. 1A). Additionally, in comparison with the untreated group, the resorption lacunae area on the distal root was reduced in the CTGF-stimulated group (Fig. 1B). After 3D reconstruction of the distal root, Fig. 1C, D shows that the resorption lacunae on the root surface decreased with CTGF administration at Day 14 and Day 28. The fuchsia area in Fig. 1C represents the resorption lacunae showing that local injection of CTGF facilitated cementum repair (Fig. 1C). In comparison with the control group, CTGF halved the volume of resorption lacunae on the root surface at Day 14 and Day 28 (Fig. 1D).

### CTGF promotes cementogenesis of hPDLSCs in a dose-dependent manner

**Fig. 2:**
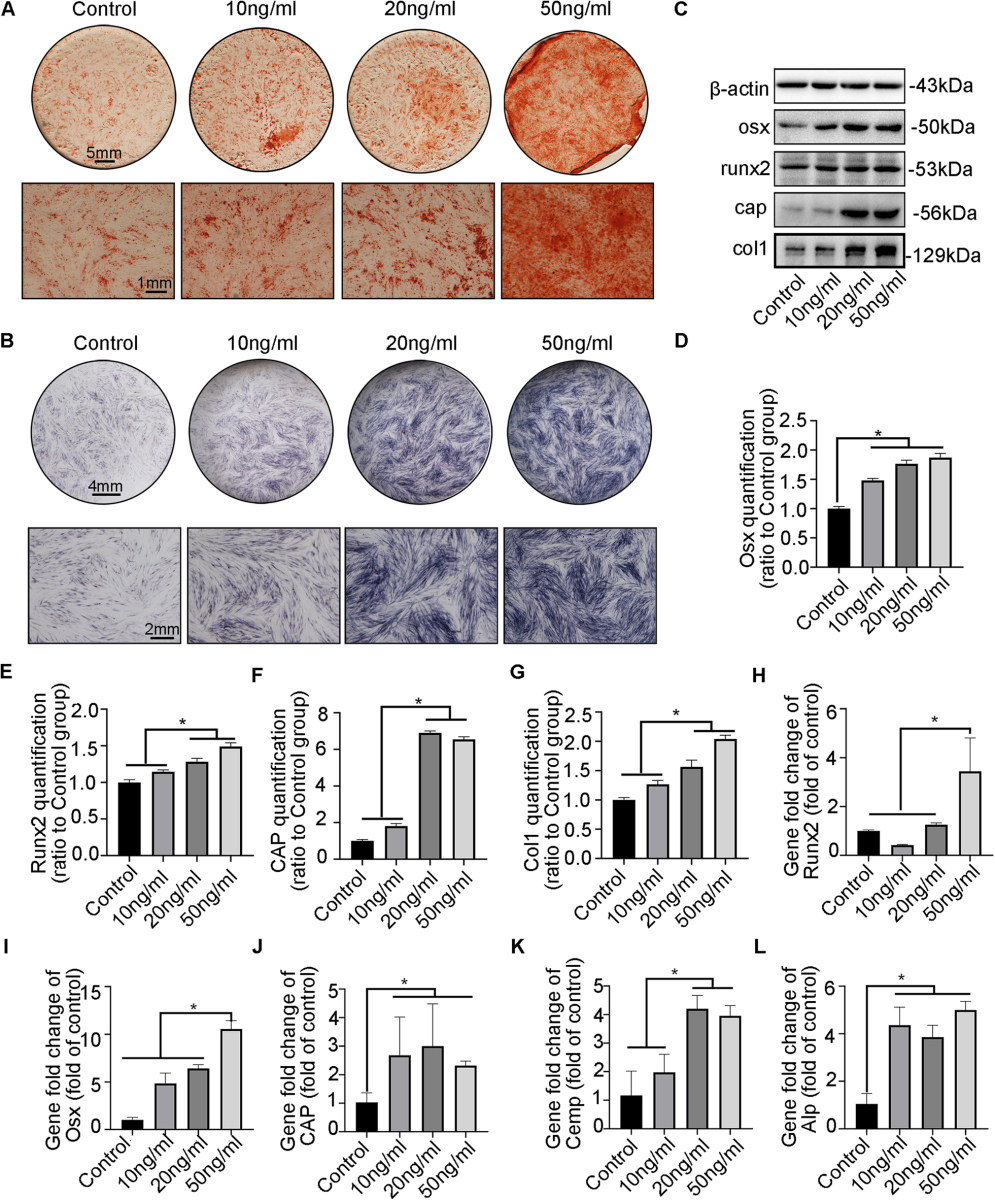
CTGF promoted cementogenesis of hPDLSCs in vitro. **A** ARS staining showed a greater increase in mineralized nodules in hPDLSCs treated with the ascending-concentration of CTGF relative to the control group on the 14th day. **B** ALP staining on day 7 demonstrated the enhanced ALP activity in hPDLSCs after CTGF treatment. **C** Representative WB results of the mineralization-related proteins showed increasing expression of Osx, Runx2, CAP and Col1 in hPDLSCs when stimulated with the concentration-ascending CTGF on the 14th day. **D**–**G** Representative charts indicating the quantification of Osx, Runx2, CAP and Col1 in Fig. 3C. *n* = 3. Data are obtained from three independent images. *p < 0.05. **H**–**L** qPCR data showed that the expression of mineralization-related factors (Runx2, Osx, CAP, CEMP, ALP) enhanced in hPDLSCs treated with the concentration-ascending CTGF on the 14th day. Data are obtained from three independent results. *p < 0.05

The stemness of hPDLSCs was identified via multidirectional differentiation of hPDLSCs and flow cytometry examination. ALP activity and ARS staining confirmed the differentiation capability of hPDLSCs (Additional file 4: Fig. S4A-E). Positive expression of STRO1 and CD146 indicated that hPDLSCs possessed the characteristics of human mesenchymal stem cells (MSC), while negative expression of CD34 and CD45 helped us distinguish hPDLSCs from haematopoietic and endothelial cells (Additional file 4: Fig. S4F-I). ARS staining revealed that mineralized nodules increased in a concentration-dependent manner (0, 10, 20, 50 ng/ml) through the combined functions of CTGF and the mineralization medium (Fig. 2A). In addition, ALP staining verified that CTGF promoted the expression of alkaline phosphatase in a concentration-dependent manner (Fig. 2B). Mineralization-related proteins or genes (Runx2, Osterix, Col1, Alp, Cap, and Cemp) were chosen based on previous research [32, 33], Additionally, WB demonstrated that CTGF enhanced the protein abundance of Runx2, Osterix, Col1, and Cap in a concentration-dependent manner (Fig. 2C), and the abundance of these targeted proteins was about twice that of the untreated group (Fig. 2D–G). Moreover, RT‒qPCR results demonstrated that the mRNA levels of Runx2, Osterix, Cap, Cemp and ALP were significantly upregulated by CTGF in a dose-dependent manner (Fig. 2H–L).

### CTGF bolsters Cx43 expression both in vivo and in vitro during cementum regeneration

**Fig. 3:**
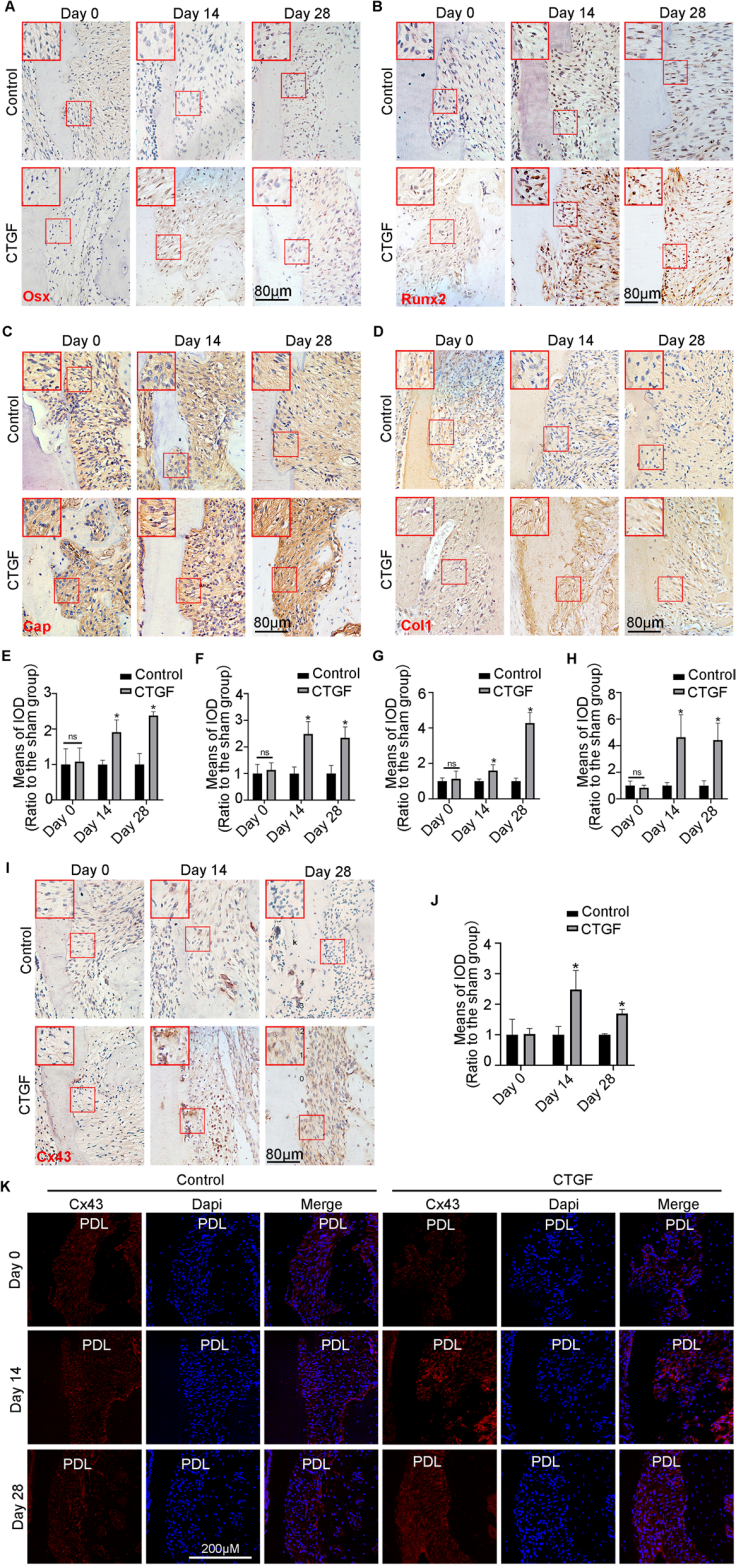
Cx43 expression was consistent with the expression of mineralization-related proteins induced by CTGF in vivo. **A**, **B** Representative IHC staining illustrated the protein expression of Osx and Runx2 with or without the treatment of CTGF on 0th day, 14th and 28th day. Images derived from three independent data. **C**, **D** Representative IHC staining illustrated the protein expression of CAP and Col1 with or without the treatment of CTGF on 0th day, 14th and 28th day. Images derived from three independent data. **E**, **F** The means of IOD of Osx and Runx2 in Fig. 4A, B. *p < 0.05. **G**, **H** The means of IOD of CAP and Cx43in Fig. 4A, B. *p < 0.05. **I** IHC results represented that the protein abundance of Cx43 with or without the treatment of CTGF on 0th day, 14th and 28th day. Images derived from three independent data. **J** The means of IOD of CAP and Cx43in Fig. 4A, B. *p < 0.05. **K** IF results represented that the abundance of Cx43 in CTGF-treated periodontal tissues. Cx43, red; nucleus, blue

**Fig. 4:**
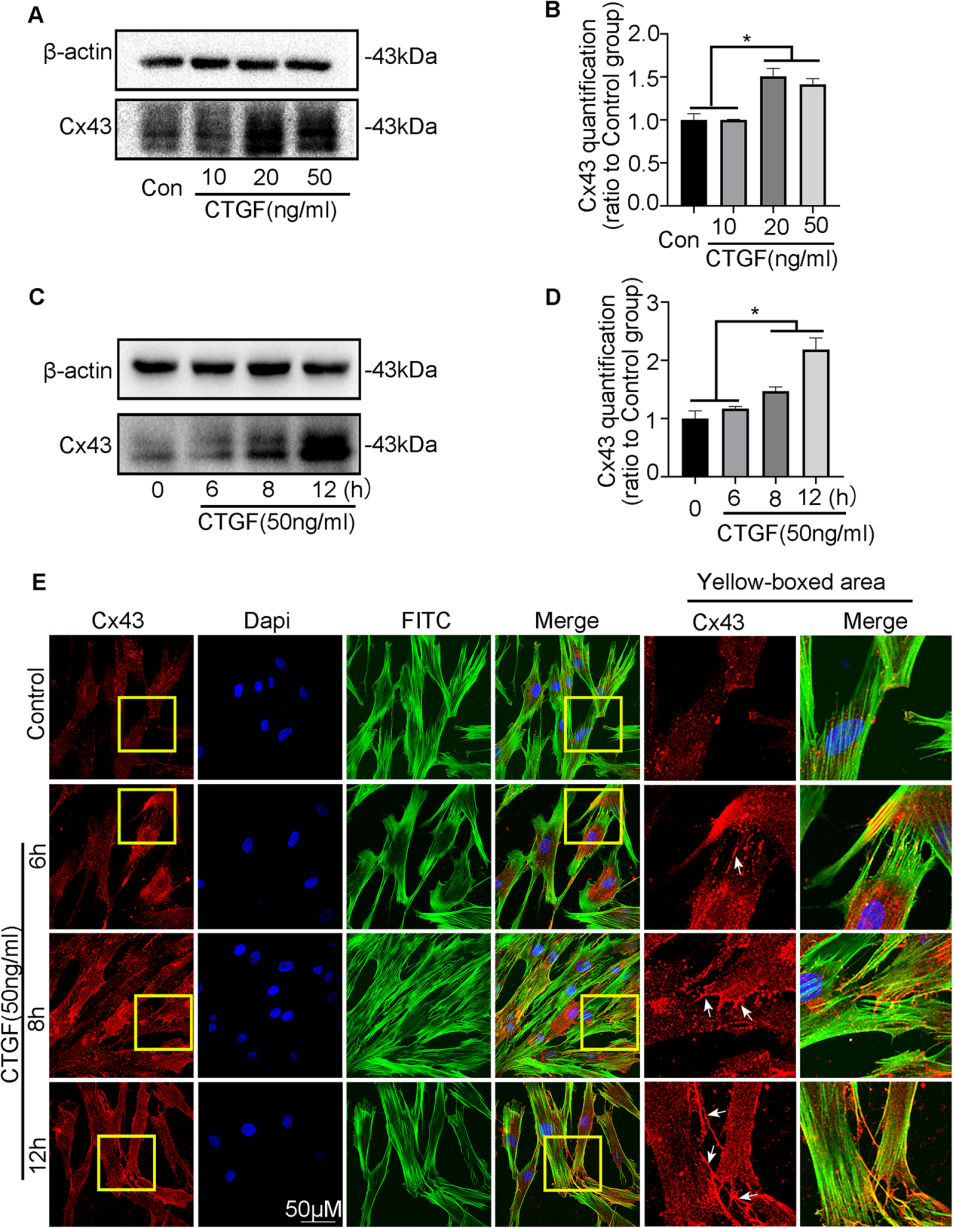
CTGF accelerated the Cx43 accumulation in time and dose-dependent manners in vitro. **A** Western blotting illustrated that CTGF accelerated the Cx43 accumulation in a concentration-dependent manners for 12 h. β-actin acted as the reference for quantification. **B** Western blotting illustrated that CTGF accelerated the Cx43 accumulation in a time-dependent manners for 12 h. β-actin acted as the reference for quantification. **C**, **D** Quantification of Cx43 proteins induced by CTGF (50 ng/ml) in Fig. 5A, B. Statistical significance is indicated. *p < 0.05. **E** IF results showed the dynamic distribution of Cx43 expression over time, and CTGF accelerated the accumulation of Cx43 at cell junctions, which manifested as red fluorescent plaques

To confirm the changes in mineralization-related protein expression during cementum regeneration, IHC was performed to observe the expression of target proteins. In comparison with the control group, we found that the nuclear-positive localization of Osterix and Runx2 was more than twofold greater under CTGF stimulation at both Day 14 and Day 28 (Fig. 3A, B, E, F). Correspondingly, the expression of Col1 and CAP was enhanced more than threefold in the CTGF-treated group (Fig. 3C, D, G, H). Interestingly, along with the enhanced expression of cementum repair-related proteins, the IHC results demonstrated that Cx43 expression in hPDLSCs from the CTGF-treated rats increased more than twofold at Day 14 and more than 0.5-fold at Day 28 compared with levels in the untreated rats (Fig. 3I, J). In addition, Cx43 immunofluorescence showed that red fluorescence-labelled Cx43 was exceedingly distributed around the root resorption craters, indicating that Cx43 might be involved in the process of cementum regeneration (Fig. 3K). The in vitro experiment showed that CTGF promoted Cx43 expression at increasing concentrations (Fig. 4A, B). At the same time, the increased expression of Cx43 was time-dependent under CTGF stimulation (Fig. 4C, D). Additionally, confocal fluorescence images showed that Cx43-labelled red fluorescence exhibited a spot-like distribution at the cell junctions and gradually accumulated with time, reaching a peak at 12 h after CTGF stimulation (Fig. 4E).

### Cx43 is a pivotal factor in CTGF-mediated cementum regeneration

**Fig. 5:**
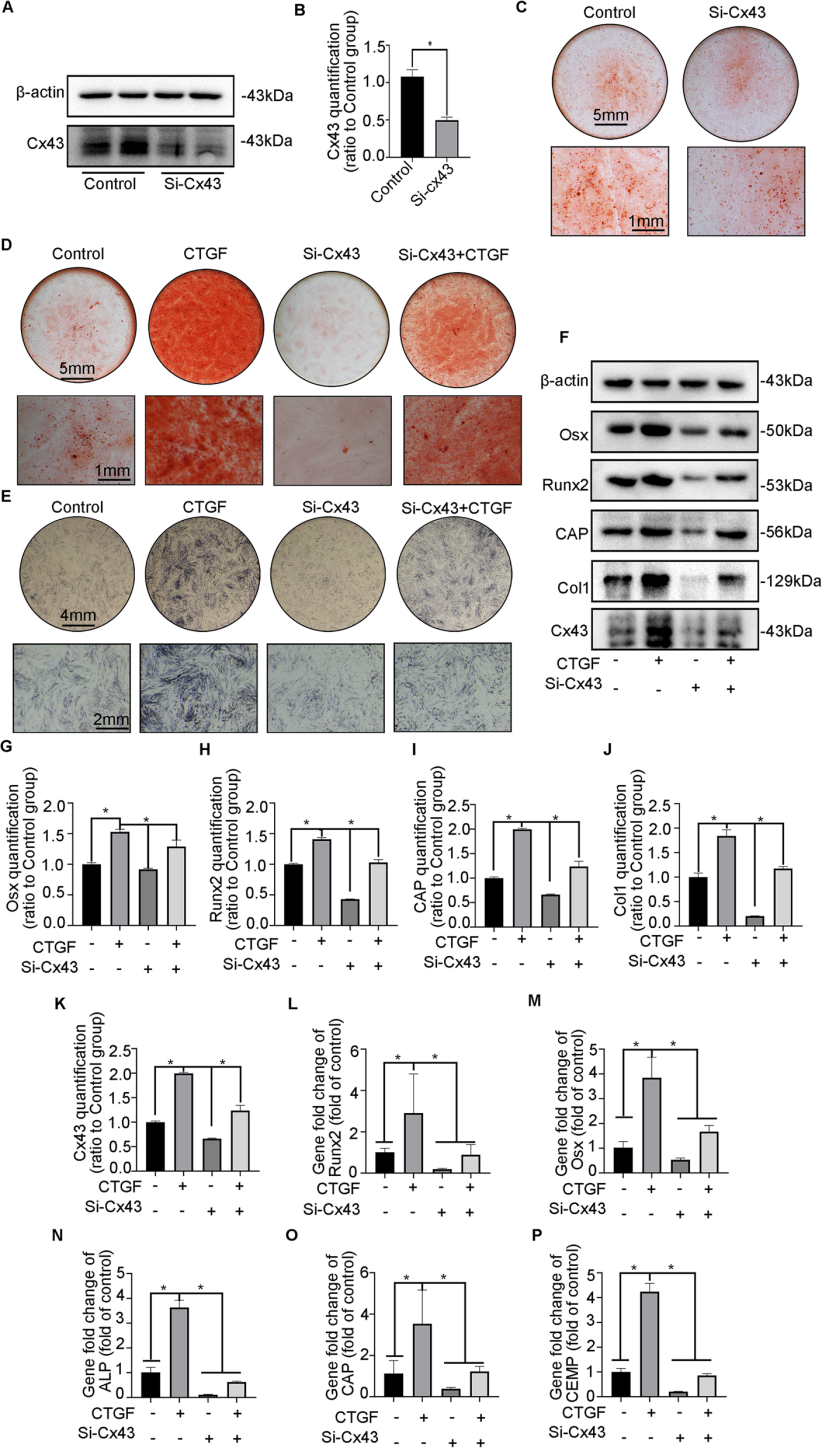
Positive cementogenesis induced by CTGF was reversed by ablation of Cx43. **A** Western blotting demonstrated the abundance of Cx43 in hPDLSCs treated with Si-Cx43. **B** Bar chart showed the levels of Cx43 in Fig. 6A ratio to the untreated groups. Data are obtained from three independent images. *n* = 3, *p < 0.05. **C** Representative images of ARS indicated the attenuated ability of mineralization induced by Si-Cx43 in hPDLSCs compared with the untreated groups. **D** Representative images of ARS staining showed the CTGF-mediated enhancement in mineralized nodules was reversed by the addition of Si-Cx43. **E** Representative images of ALP staining showed the CTGF-mediated enhancement in ALP activity was reversed by Si-Cx43. **F** Representative images of the data of mineralization-associated proteins (Runx2, Osx, CAP, Col1) and Cx43 obtained by western blotting showed that increased mineralization capacity of CTGF expression was reverted by Si-Cx43. **G**–**K** Bar chart showed the levels of Runx2, Osx, CAP, Col1 and Cx43 in Fig. 6F ratio to the untreated control. Data are obtained from three independent images. *n* = 3, *p < 0.05. **L–P** mRNA expression of Runx2, Osx, ALP, CAP and CEMP, as detected by PCR (*n* = 3) as detected by PCR

To further determine whether Cx43 plays a critical role in CTGF-mediated cementum repair, we applied the small interfering RNA Si-Cx43 in hPDLSCs for mineralized detection. Firstly, WB results showed that Si-Cx43 significantly reduced the expression of Cx43 (Fig. 5A, B). Subsequently, ARS staining showed that the application of Si-Cx43 to the mineralization medium reduced the formation of mineralized nodules (Fig. 5C). Therefore, we co-stimulated hPDLSCs with Si-Cx43 and CTGF to observe their impact on mineralization. ARS staining suggested that CTGF enhanced the mineralization capacity of hPDLSCs, which was reversed by the addition of Si-Cx43 (Fig. 5D). Similarly, ALP activity was significantly upregulated by CTGF, which was inhibited by Cx43 knockdown (Fig. 5E). In addition, the expression of mineralization-related proteins was enhanced after CTGF treatment and was inhibited by Cx43 ablation with Si-Cx43 (Fig. 5F–J). The Cx43 expression tendency was consistent with that of the above mineralization-related proteins (Fig. 5K). Furthermore, CTGF upregulated mineralization-related genes, and the addition of Si-Cx43 impaired the mineralization tendency induced by CTGF (Fig. 5L–P).

**Fig. 6:**
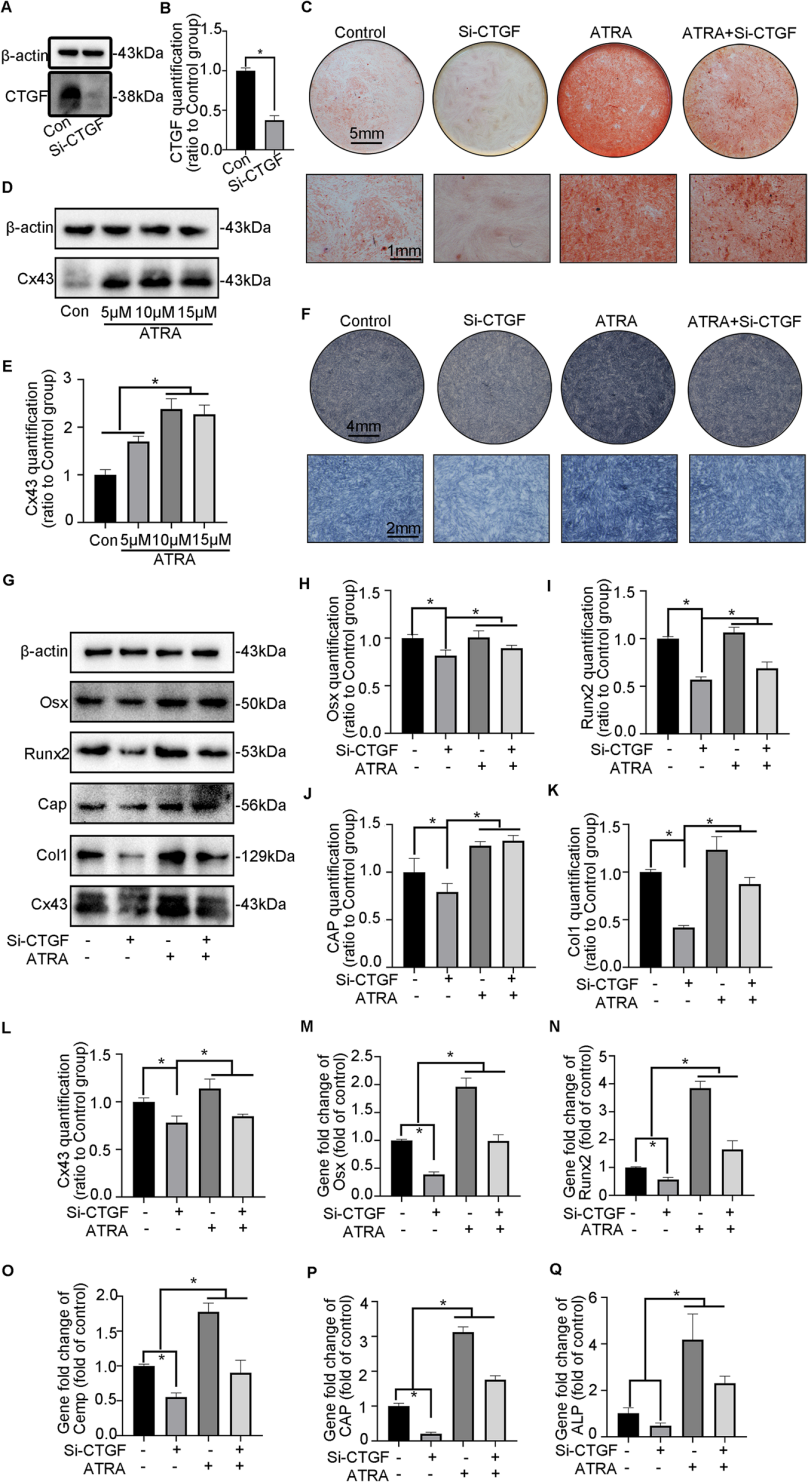
Negative cementogenesis induced by Si-CTGF in hPDLSCs was motivated by the Cx43 agonist. **A** Western blotting demonstrated the abundance of CTGF in hPDLSCs treated with Si-CTGF. **B** Bar chart showed the levels of CTGF in** A** ratio to the untreated groups. Data are obtained from three independent images. *n* = 3, *p < 0.05. **C** Western blotting demonstrated the abundance of Cx43 in hPDLSCs treated with Cx43 agonist ATRA. **D** Bar chart showed the levels of Cx43 in** C** ratio to the untreated groups. Data are obtained from three independent images. *n* = 3, *p < 0.05. **E** Representative images of ARS indicated the attenuated ability of mineralization induced by Si-CTGF in hPDLSCs was regained by Cx43 agonist ATRA when compared with the untreated groups. **F** Representative images of ALP staining showed the attenuated ALP activity stimulated by Si-CTGF in hPDLSCs was regained by Cx43 agonist ATRA when in comparison to the untreated groups. **G** Representative image of the data of mineralization-associated proteins (Runx2, Osx, CAP, Col1) and Cx43 obtained by western blotting showed that the decreased mineralization capacity of CTGF expression was reverted by Cx43 agonist ATRA. **H**, **I** Bar chart showed the protein levels of Runx2, Osx, CAP, Col1 and Cx43 in **G** ratio to the untreated groups. Data are obtained from 3 independent images. *n* = 3, *p < 0.05. **J** mRNA expression of Osx, Runx2, ALP, CAP and CEMP, as detected by PCR (*n* = 3) as detected by PCR

On the other hand, we continued to explore the mechanism of CTGF-mediated cementoblast differentiation by applying the Cx43 agonist ATRA together with the small interfering RNA Si-CTGF. Firstly, Si-CTGF was confirmed to decrease the expression of CTGF in hPDLSCs (Fig. 6A, B). Additionally, ATRA enhanced the expression of Cx43 in a concentration-dependent manner (Fig. 6C, D). On this basis, we applied ATRA to hPDLSCs after the ablation of CTGF. ARS staining showed that Si-CTGF reduced the mineralization capacity of hPDLSCs, while ATRA reversed the inhibitory effect of Si-CTGF (Fig. 6E). ALP staining demonstrated that ALP activity was attenuated after Si-CTGF treatment and was restored by ATRA treatment (Fig. 6F). At the same time, WB results showed that Si-CTGF decreased the expression of mineralization-related proteins (Osx, Runx2, CAP, Col1), which was reversed by the application of ATRA (Fig. 6G–K). The Cx43 expression tendency was consistent with that of the above mineralization-related proteins (Fig. 6G, I). Further PCR studies showed that Si-CTGF inhibited the expression of mineralization-related genes, while the addition of ATRA reversed the inhibitory effect induced by Si-CTGF. The Cx43 expression tendency was consistent with that of the abovementioned mineralization-related proteins (Fig. 6J).

### CTGF promotes cementogenesis through the Cx43/β-catenin axis

**Fig. 7:**
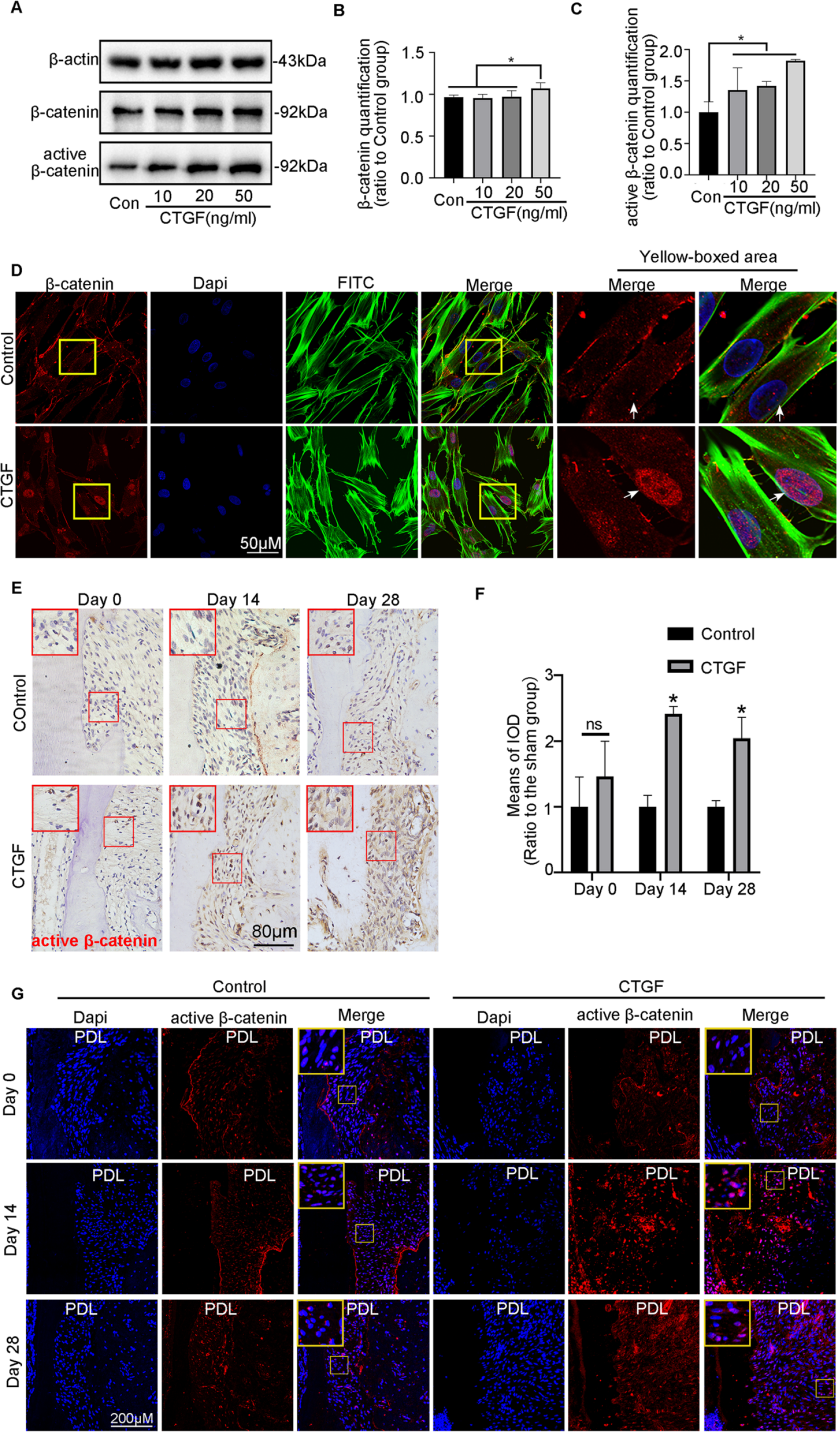
CTGF triggered β-catenin signalling in dose-dependent manners. **A** Western blotting demonstrated the abundance of β-catenin and active β-catenin in hPDLSCs treated with the concentration-ascending CTGF (0, 10, 20, 50 ng/ml). **B**, **C** Bar chart showed the protein abundance of β-catenin and active β-catenin in** A** ratio to the untreated control. Data are obtained from three independent images. *n* = 3, *p < 0.05. **D** Representative IF images of β-catenin staining, in hPDLSCs that were untreated, treated with CTGF. **E** Representative IHC staining illustrated that the abundance of active β-catenin in CTGF-treated hPDLSCs. active β-catenin, red; nucleus, blue; green, cytoskeleton. **F** A bar chart represented the means of IOD of active β-catenin. *p < 0.05. **G** Representative IF staining illustrated that the abundance of active β-catenin in CTGF-treated periodontal tissues. active β-catenin, red; nucleus, blue

**Fig. 8:**
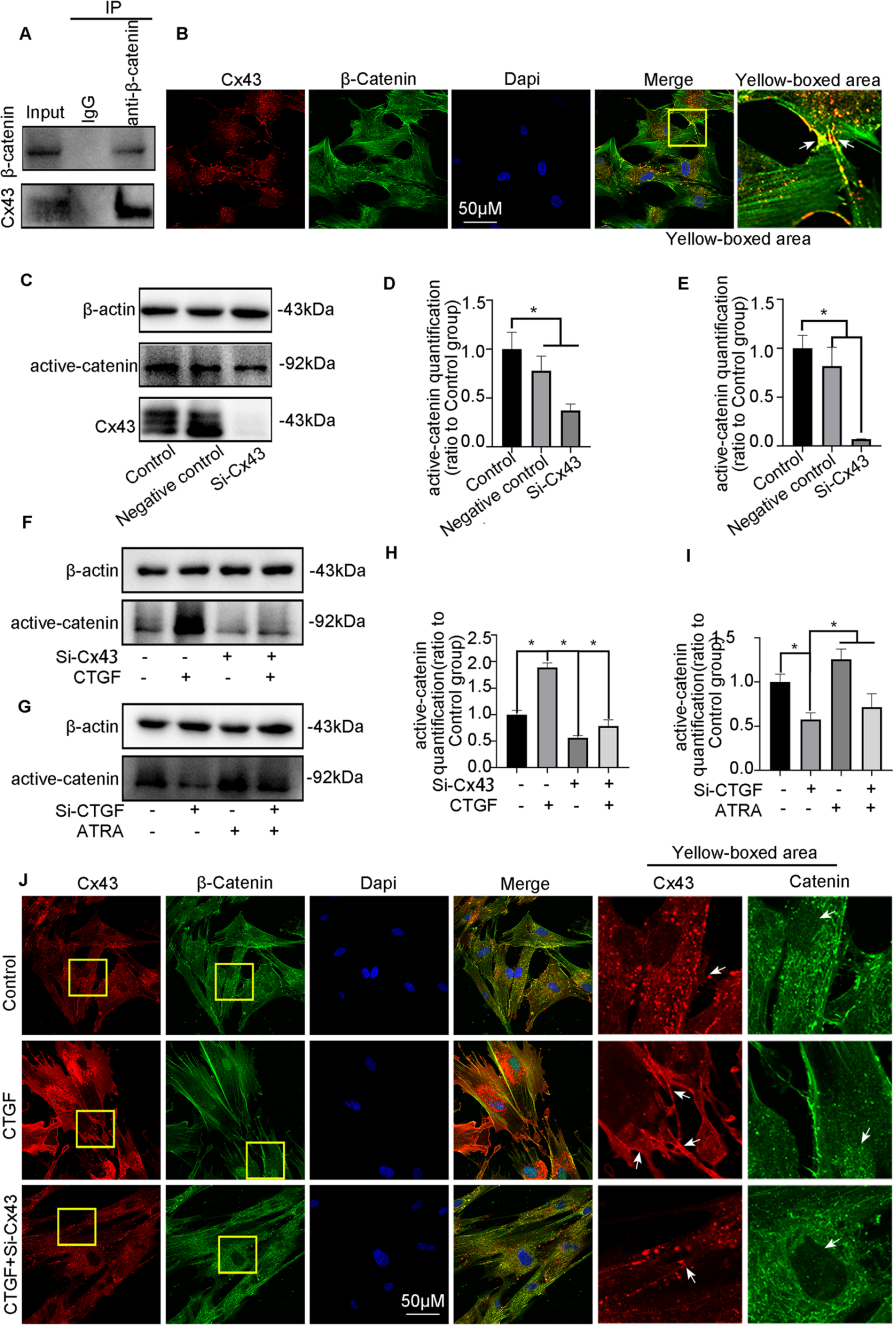
CTGF triggered Cx43/β-catenin signalling to promote cementogenesis. **A** Co-IP experiments indicated the β-catenin antibody could interact with Cx43 protein. **B** Double fluorescence staining methods showed Cx43 (Cx43) and β-catenin (green) accumulated at the membranes and cell junctions of hPDLSCs. **C** Western blotting demonstrated the abundance of the active β-catenin and Cx43 in hPDLSCs transfected with Si-Cx43. **D**, **E** Bar chart demonstrated the abundance of Cx43 and active β-catenin in Fig. 8C ratio to the untreated control. Data are obtained from three independent images. *n* = 3, *p < 0.05. **F** Western blot for the abundance of the active β-catenin in hPDLSCs treated with Si-Cx43 and CTGF (50 ng/ml). **G** Western blot for the abundance of the active β-catenin in hPDLSCs treated with Cx43 agonist ATRA and Si-CTGF. **H**, **I** Bar chart shows the protein levels of active β-catenin in Fig. 8F, G ratio to the untreated groups. Data are obtained from three independent images. *n* = 3, *p < 0.05. **J** Double fluorescence staining methods showed Cx43 (red) accumulated at cell junctions and β-catenin (green) translocated into cell nucleus under the stimulation of CTGF (50 ng/ml), this effect was reverted by Si-Cx43

A previous study confirmed that PDL progenitor cells with constitutively activated β-catenin promote cementum growth [5]. Thus, we examined whether β-catenin is closely related to the mechanism of CTGF-mediated cementogenesis. Figure 7A–C shows that CTGF promoted the expression of active β-catenin in a dose-dependent manner at 8 h and that total β-catenin expression increased slightly after 50 ng/ml CTGF treatment. Immunofluorescence results demonstrated that β-catenin translocated into the nucleus after the addition of CTGF (Fig. 7D). Additionally, in vivo studies revealed that CTGF induced significant β-catenin nuclear staining during cementum repair, particularly on Day 14 (Fig. 7E, F). Furthermore, immunofluorescence staining of periodontal tissue revealed that CTGF promoted strong red fluorescence-labelled β-catenin expression in the nucleus at Day 14 (Fig. 7G). To further determine whether Cx43 interacts with β-catenin, we performed a co-IP experiment, which confirmed a protein–protein interaction between Cx43 and β-catenin (Fig. 8A). Next, we performed double staining and examined red fluorescence-labelled Cx43 and green fluorescence-labelled β-catenin localization. Fluorescence staining showed that Cx43 and β-catenin were relatively uniformly distributed on the cell membrane and at cell junctions, suggesting that there might be an interactive relationship between these two proteins (Fig. 8B). Then, WB experiments indicated that the abundance of active β-catenin was reduced in the presence of Si-Cx43 (Fig. 8C–E). Additionally, the strong expression of β-catenin induced by CTGF was abolished by Si-Cx43 (Fig. 8F, G). On the other hand, ablation of CTGF inhibited the activation of β-catenin, and the Cx43 agonist ATRA rescued the lower β-catenin expression in the Si-CTGF group (Fig. 8H, I). Furthermore, double fluorescence staining showed that the red fluorescence-labelled Cx43 was enhanced in the CTGF group, accompanied by enhanced nuclear localization of green fluorescence-labelled β-catenin. After si-Cx43 application, the Cx43 expression induced by CTGF decreased, accompanied by a reduction in nuclear β-catenin expression (Fig. 8J). Overall, there may be a protein interaction between Cx43 and β-catenin, and the strong membrane distribution of Cx43 induced by CTGF promotes the nuclear localization of β-catenin. Additionally, we validated the role of β-catenin in CTGF-mediated cementogenesis by using the β-catenin activator LiCl to promote β-catenin activation. First, WB results showed that LiCl successfully promoted the expression of activated β-catenin (Additional file 5: Fig. S5A-B). Subsequently, ALP staining and PCR results showed that Si-Cx43 attenuated CTGF-mediated ALP activity and mineralization-related gene expression, and the addition of LiCl successfully reversed this effect (Additional file 5: Fig. S5C-H).

## Discussion

Cementum regeneration is an important part of periodontal tissue regeneration, and overcoming this daunting challenge and finding new therapies is difficult. Periodontal ligament cells contribute to the repair of root resorption resulting from orthodontic forces by acting as a source of cementum regeneration [30]. At present, other studies have reported that the application of cytokines was helpful for cementum regeneration [7]. TGFβ-3, a novel cytokine that promotes expression of the cementoblast-specific protein CEMP, is involved in cementum regeneration [34]. CTGF is closely related to TGF-β signalling, and CTGF-labelled stem cells in the trabecular bone area have been found to differentiate in multiple directions [35, 36]. Previous studies have demonstrated that CTGF facilitated osteogenic differentiation of osteoblasts and periodontal regeneration. However, its effect on the cementogenesis of hPDLSCs is unknown, and the underlying mechanism is unclear [9, 37]. Our experiments confirmed that CTGF reduced the resorption lacunae of the cementum root surface. In addition, CTGF was demonstrated to promote the mineralization of hPDLSCs in vitro by upregulating mineralization-related markers (Runx2, ALP, Osx and Col1) and cementum-specific markers (CAP, CEMP). The innovative finding of our study is that CTGF promotes cementum repair of root resorption and cementogenesis.

At present, the mechanism by which CTGF regulates cementum formation is not fully understood. Cx43 has been widely studied in the process of tooth development [16]. Intercellular communication mediated by Cx43 can promote the differentiation of ameloblasts and exhibits significant effects on enamel mineralization [38]. Our previous studies showed that Cx43 was a downstream target protein of CTGF in chondrocytes that regulated cell-to-cell communication [27, 31]. Simultaneously, cementocytes specifically express Cx43 as a gap junction carrier to facilitate intercellular communication [20]. Therefore, we speculated that Cx43 regulated by CTGF was involved in the mechanism of cementogenesis. In vivo results demonstrated that the upregulation of Cx43 accompanied the enhancement of mineralized protein in CTGF-regulated cementum repair. In vitro experiments verified that CTGF promoted the expression of Cx43. Furthermore, CTGF-mediated cementoblast differentiation was inhibited by Cx43 knockdown in vitro. The inhibited mineralization capacity caused by Si-CTGF was stimulated by the Cx43 agonist ATRA. Therefore, we have reason to conclude that Cx43 may be the key factor in CTGF-mediated cementogenesis. Additionally, the Wnt/β-catenin signalling pathway exerts significant effects in regulation of multiple organ functions [5, 21]. Positive Wnt1 expression was found to significantly contribute to cementum development in a time-dependent manner [39]. Consistently activating β-catenin in Axin^+^ or Gli1^+^ periodontal ligament cells caused cementum hyperplasia [5, 40]. Subsequently, loss of function of β-catenin inhibited cementum growth [5]. As for cementum regeneration, Li^+^ ions promoted cementum differentiation and regeneration by activating the Wnt/β-catenin pathway [41]. When periodontal defects occur, local addition of lithium chloride or construction of a lentivirus overexpressing β-catenin can promote cementum repair [42]. The Wnt/β-catenin pathway is also closely related to the mechanism of root resorption and repair [43]. Our data confirmed that CTGF enhanced the protein abundance of active β-catenin and induced the nuclear translocation of β-catenin, suggesting that β-catenin might be involved in cementum regeneration. Furthermore, both Cx43 and β-catenin were expressed at the cell membrane and cell junctions, indicating similar distributions and functions [23]. The bone cortex of both osteocyte-specific Cx43 and β-catenin conditional ablation mice showed the characteristics of thinning of the bone cortex and more voids [18]. Moreover, Cx43 expression was highly correlated with β-catenin expression in primary osteoblasts and bone marrow stromal cells. Thus, we speculate that β-catenin acts as a downstream protein of Cx43 to regulate CTGF-mediated cementoblast differentiation. Besides, Cx43 was shown to interact with β-catenin protein. Ablation of Cx43 reduced CTGF-mediated β-catenin activation and reversed the nuclear translocation of β-catenin induced by CTGF. Cx43 activation invalidated the low expression of the β-catenin pathway induced by Si-CTGF. Additionally, the β-catenin agonist LiCl reversed the inhibitory effect of Si-Cx43 on hPDLSCs cementoblast differentiation in the presence of CTGF. In summary, we believe that β-catenin acts as a downstream target of Cx43 to regulate cementogenesis, which is consistent with previous studies [23, 24].

## Conclusions

**Fig. 9:**
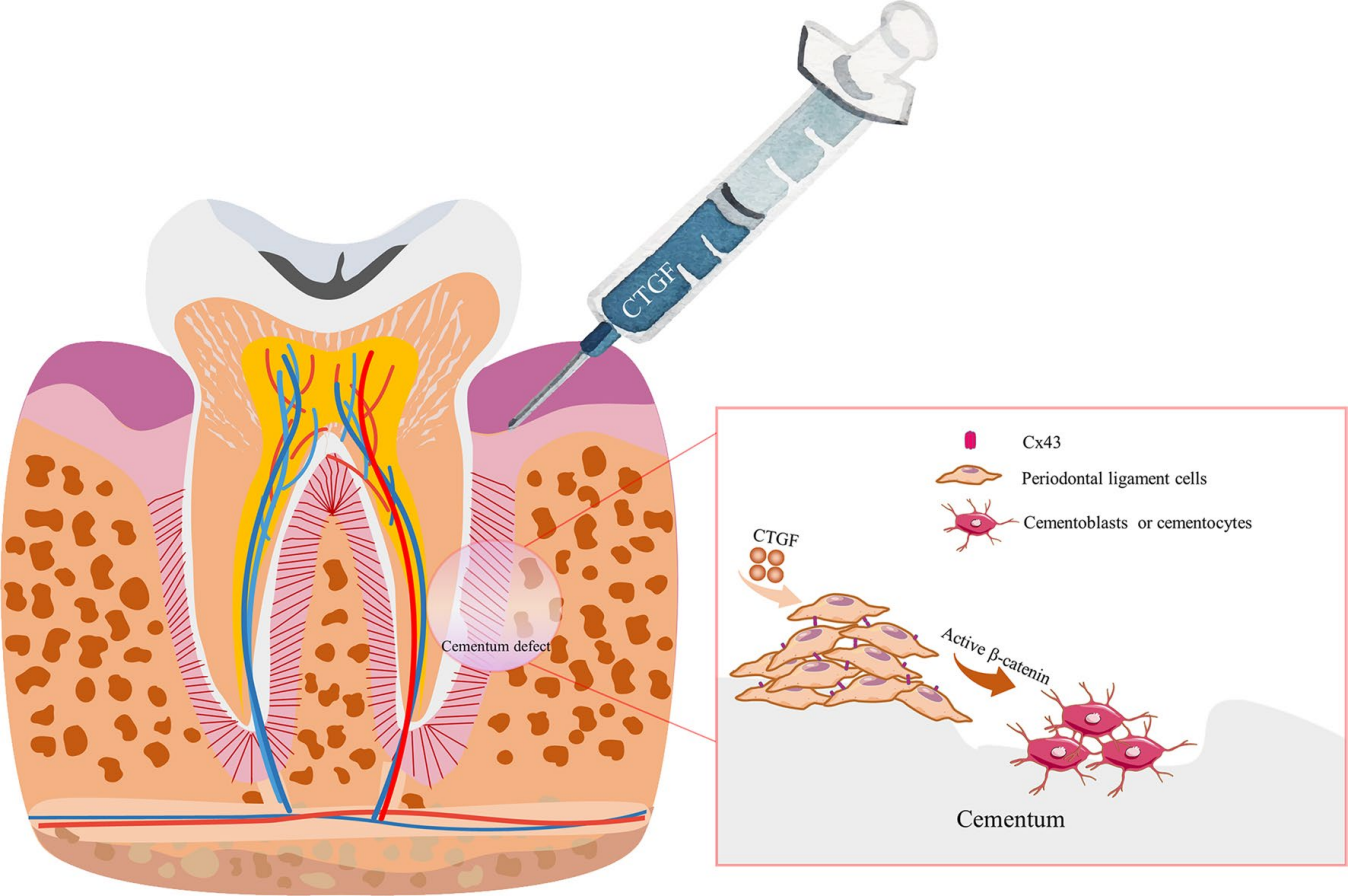
CTGF promoted cementum repair and cementogenesis of PDL cells through activation of the Cx43/β-catenin signalling axis

Collectively, our data demonstrate that CTGF promotes cementum regeneration and cementum repair of root resorption craters. The Cx43/β-catenin signalling axis mediated CTGF-regulated cementum regeneration, and the enhancement of this signalling axis contributed to cementum regeneration and it might be a possible therapeutic target for OIRR (Fig. 9) [44].

## Supplementary Information

**Additional file 1. Figure S1**. The Ethics Committee of West China Stomatological Hospital for animal study.

**Additional file 2. Figure S2**. Establishment of tooth root resorption model and three-dimensional (3D) reconstruction of tooth root.

**Additional file 3. Figure S3**. The Ethics Committee of West China Stomatological Hospital for human study.

**Additional file 4. Figure S4**. IhPDLSCs morphology and multilineage differentiation ability.

**Additional file 5. Figure S5**. Si-Cx43 attenuates CTGF-mediated cementoblast differentiation, and β-catenin agonists reverse this effect.

**Additional file 6.** Sequences of primer pairs of housekeeping and mineralization-related genes in hPDLSCs for qPCR.

## Data Availability

No publicly available data or shared data are cited.

## Abbreviations

OIRR: Orthodontic-induced external root resorption
hPDL: Human periodontal ligament
hPDLSCs: Human periodontal ligament stem cells
CTGF: Connective tissue growth factor
CCN2: Cellular communication network factor 2
Cx43: Connexin43
ALP: Alkaline phosphatase
ARS: Alizarin red
WB: Western blotting
RT-PCR: Quantitative RT-PCR
Cap: Cementum attachment proteins
Cemp: Human cementum protein
ATRA: All-trans-retinoic acid
Co-IP: Co-immunoprecipitation
