# Supplementary figures and images for: Connective tissue growth factor promotes cementogenesis and cementum repair via Cx43/β-catenin axis
Source: Stem Cell Res Ther. 2022 Sep 6;13:460. doi: 10.1186/s13287-022-03149-8 (PMC9450312; doi:10.1186/s13287-022-03149-8)

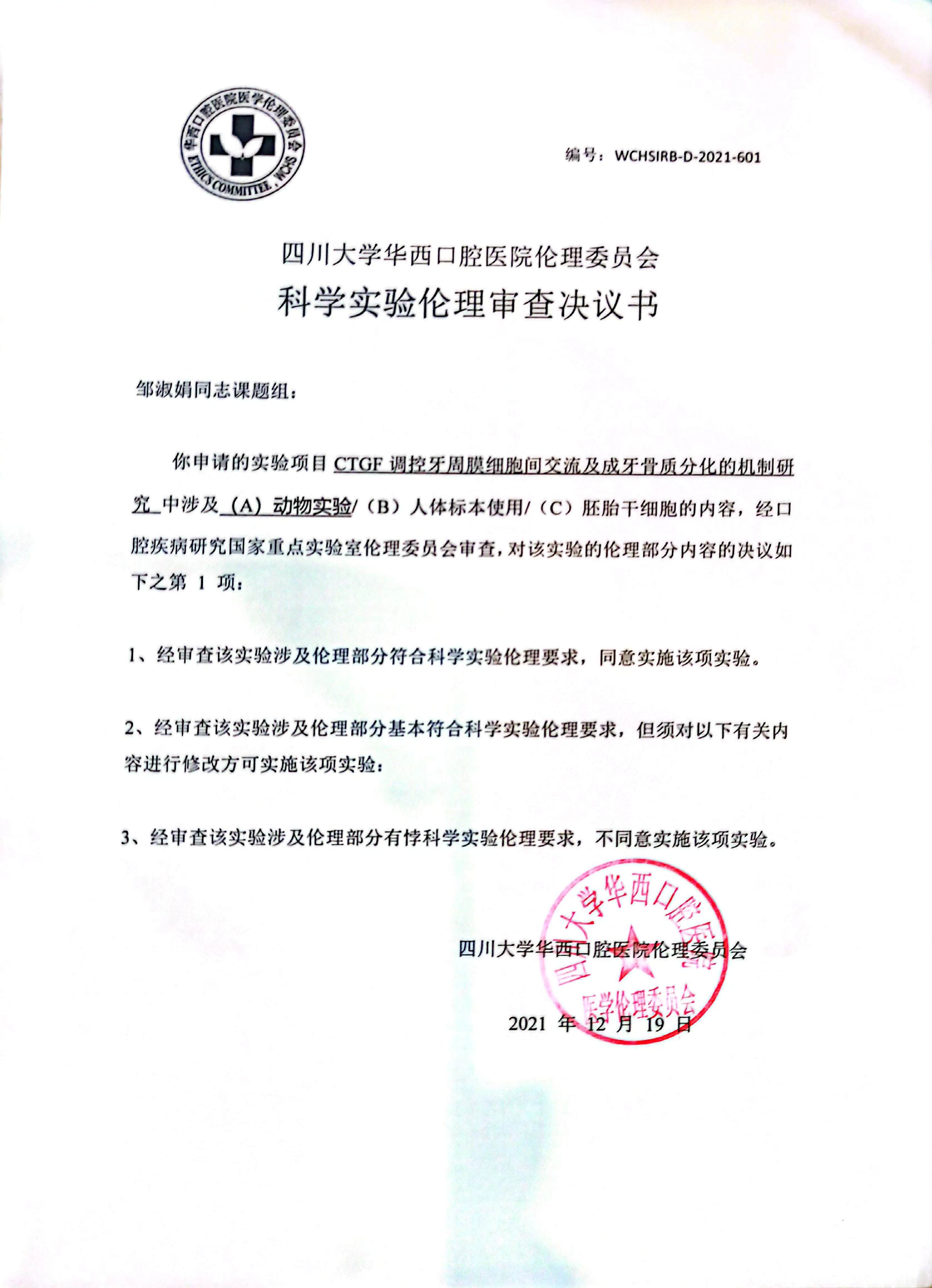

Supplement: Supplementary file 1 — Additional file 1. Figure S1. The Ethics Committee of West China Stomatological Hospital for animal study. [file 13287_2022_3149_MOESM1_ESM.docx]

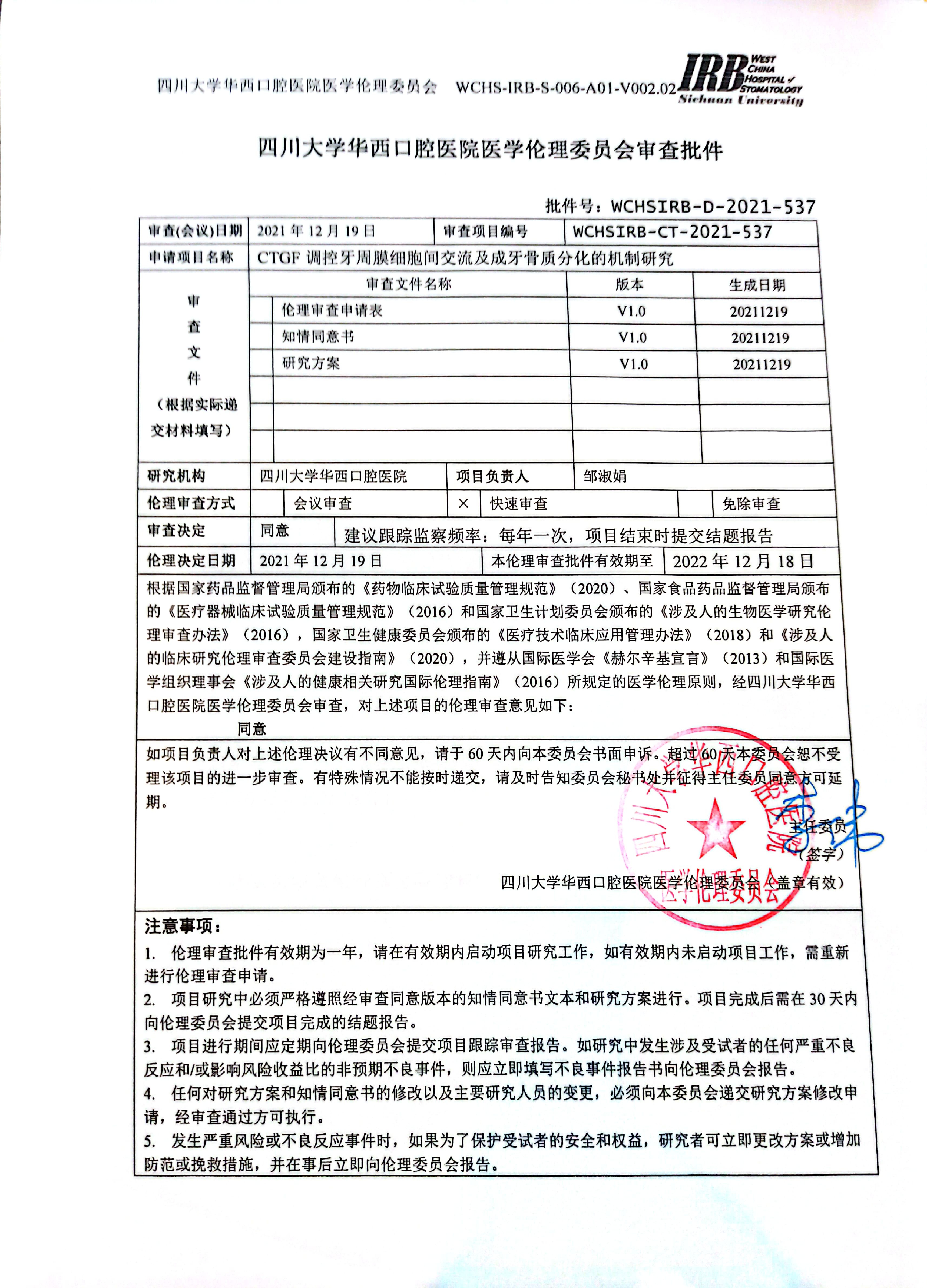

Supplement: Supplementary file 3 — Additional file 3. Figure S3. The Ethics Committee of West China Stomatological Hospital for human study. [file 13287_2022_3149_MOESM3_ESM.docx]
