# Supplementary material for: Connective tissue growth factor promotes cementogenesis and cementum repair via Cx43/β-catenin axis
Source: Stem Cell Res Ther. 2022 Sep 6;13:460. doi: 10.1186/s13287-022-03149-8 (PMC9450312; doi:10.1186/s13287-022-03149-8)
Supplement: Supplementary file 2 — Additional file 2. Figure S2. Establishment of tooth root resorption model and three-dimensional (3D) reconstruction of tooth root. [file 13287_2022_3149_MOESM2_ESM.docx]

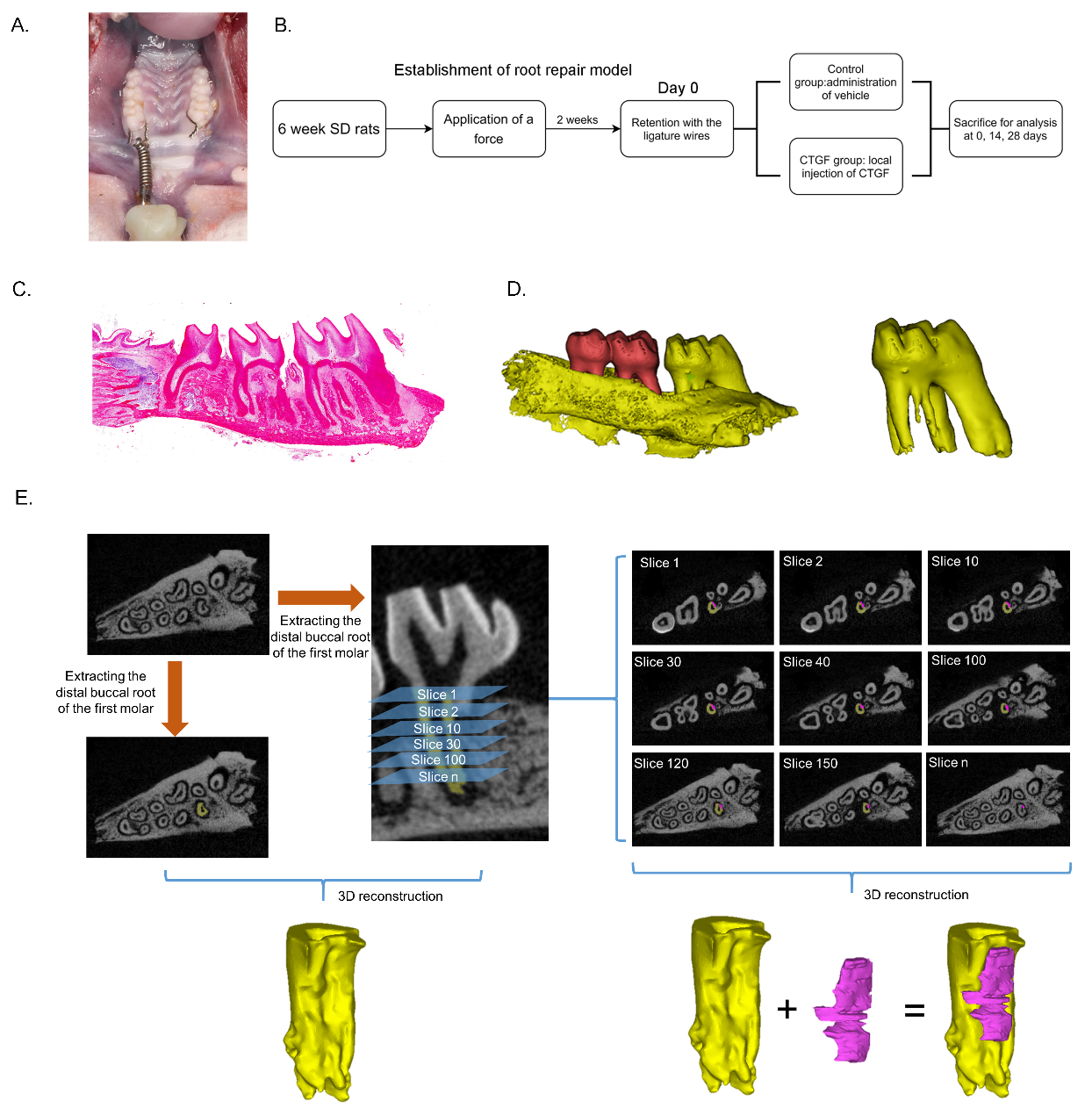


**Additional file 2 figure S2. Establishment of tooth root resorption model and three-dimensional (3D) reconstruction of tooth root.**

**a.** Installation of the intraoral orthodontic appliance.

**b.** Flow chart of animal experiment design.

**c.** HE staining showing the tooth movement distance.

**d.** Reconstruction of the left maxilla by mimics 21.

**e.** Simulate the root defect area by mimics 21. The yellow area represents the existing root and the fuchsia represents root lacunae area. Reconstructing the distal buccal root by suitable threshold selection, which is built up by the yellow threshold. Then, according to the original shape of the root, the fuchsia threshold was assigned to each layer of the root resorption lacunae (slice1 to slice n) in the two-dimensional cross-section, and the reportion lacunae was reconstructed by the fuchsia labelled area in every two-dimensional slices.
