# Supplementary material for: Connective tissue growth factor promotes cementogenesis and cementum repair via Cx43/β-catenin axis
Source: Stem Cell Res Ther. 2022 Sep 6;13:460. doi: 10.1186/s13287-022-03149-8 (PMC9450312; doi:10.1186/s13287-022-03149-8)
Supplement: Supplementary file 4 — Additional file 4. Figure S4. IhPDLSCs morphology and multilineage differentiation ability. [file 13287_2022_3149_MOESM4_ESM.docx]

**
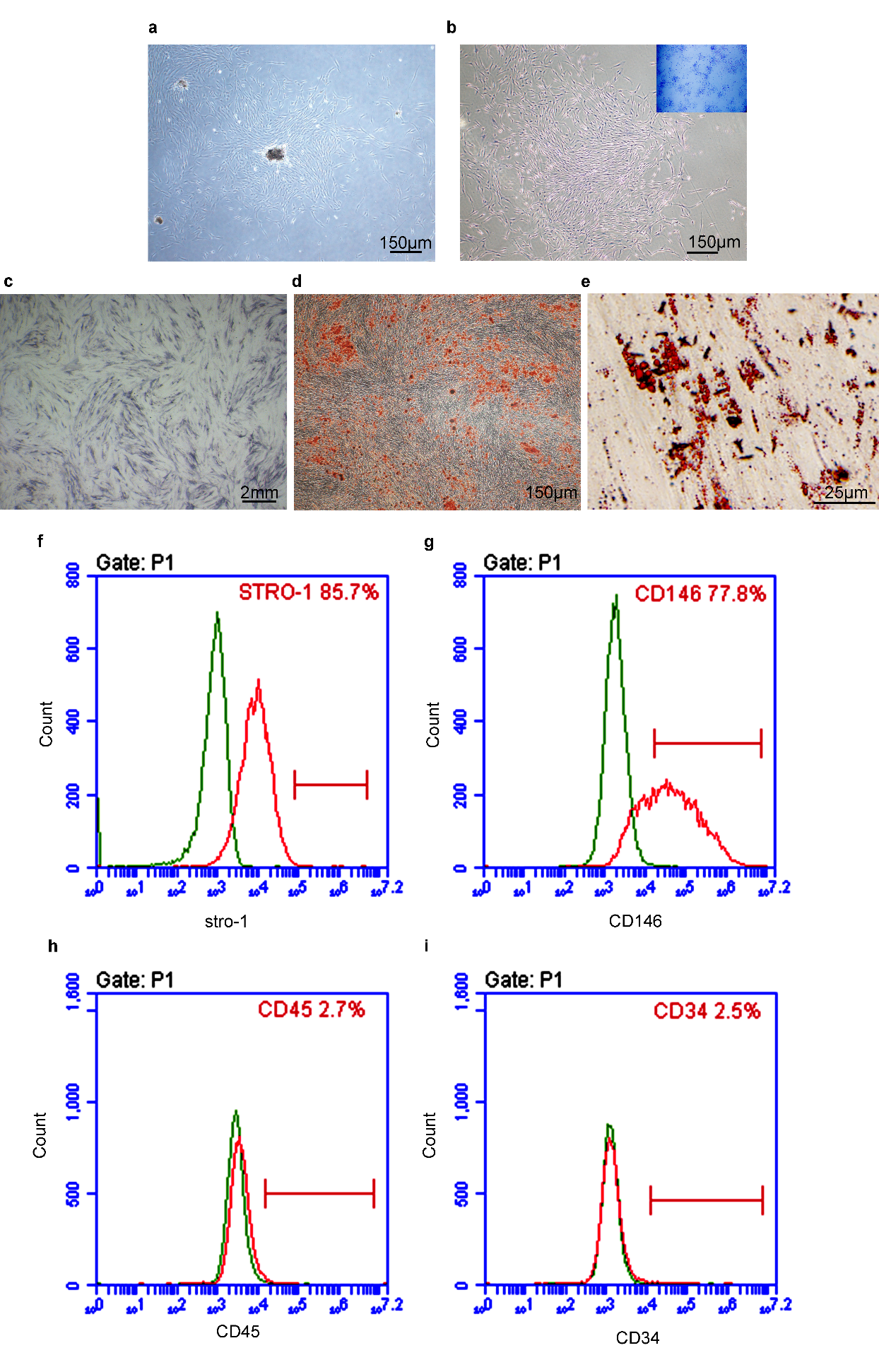
**

**Additional file 4 figure S4. hPDLSCs morphology and multilineage differentiation ability**

**a.** Extracted third molars and PDL from the middle-third root surfaces was isolated. Primary cells typically grew around the tissue mass and multiplied.

**b.** Single colonies formed and then PDLSCs were stained with crystal violet.

**c-d.** ALP staining (C) and Alizarin Red staining (D) was performed to identify osteogenic differentiation capacity of PDLSCs.

**e.** Oil Red O staining was performed to access the adipogenesis capacity of PDLSCs.

**h-i.** Flow cytometry data showed the positive expression for STRO-1 and CD146, and the negative expression for CD34 and CD45 in hPDLSCs

Detailed methods of single colonies experiments, osteogenic differentiation and adipogenesis differentiation was in reference to previous study^1,2,3^.

1. Xie J, Zhang D, Zhou C, Yuan Q, Ye L, Zhou X. Substrate elasticity regulates adipose-derived stromal cell differentiation towards osteogenesis and adipogenesis through β-catenin transduction. *Acta biomaterialia.* 2018;79:83-95.

2. Seo B, Miura M, Gronthos S, et al. Investigation of multipotent postnatal stem cells from human periodontal ligament. *Lancet (London, England).* 2004;364(9429):149-155.

3. Xi X, et al. Nrf2 activation is involved in osteogenic differentiation of periodontal ligament stem cells under cyclic mechanical stretch. *Exp. Cell Res.* **403**,112598.(2021).
