# Supplementary material for: Connective tissue growth factor promotes cementogenesis and cementum repair via Cx43/β-catenin axis
Source: Stem Cell Res Ther. 2022 Sep 6;13:460. doi: 10.1186/s13287-022-03149-8 (PMC9450312; doi:10.1186/s13287-022-03149-8)
Supplement: Supplementary file 5 — Additional file 5. Figure S5. Si-Cx43 attenuates CTGF-mediated cementoblast differentiation, and β-catenin agonists reverse this effect. [file 13287_2022_3149_MOESM5_ESM.docx]

**
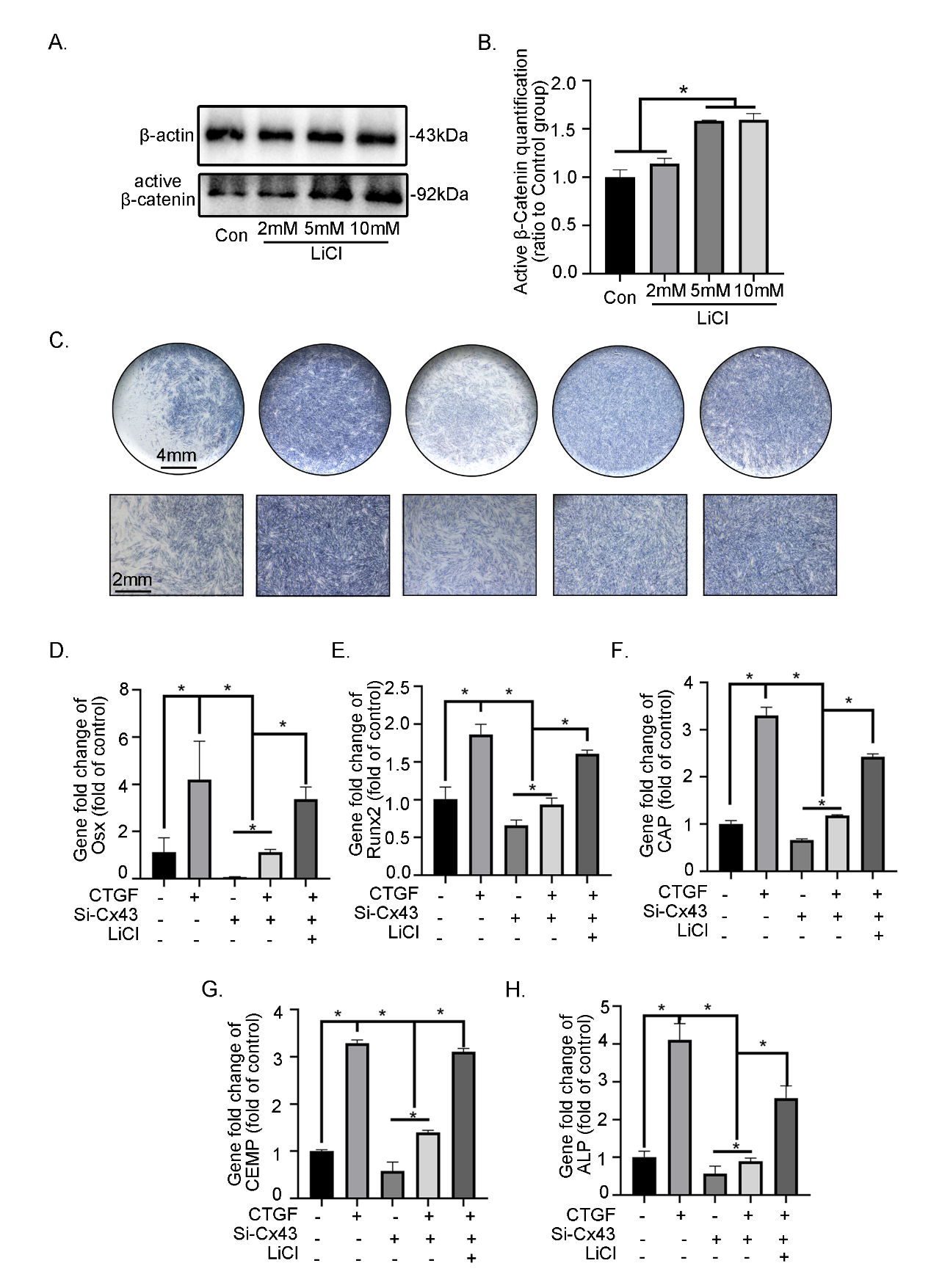
**

**Additional file 5 figure S5.** Si-Cx43 attenuates CTGF-mediated cementoblast differentiation, and β-catenin agonists reverse this effect

A. The WB band indicates the expression of active β-catenin after LiCl stimulation in hPDLSCs. B. Quantitative analysis of active β-catenin protein levels in supplemental Fig.3.a, *, P < 0.05.

C. Representative ALP staining showed that the CTGF group (50ng/ml) increases the ALP activity of hPDLSCs when compared with the control group, and the Si-Cx43 and CTGF co-intervention group had lower alkaline phosphatase activity than the CTGF group alone, β-catenin agonist LiCl (5mM) reversed the above effect.

D-H. PCR results showed the mRNA expression levels of mineralization-related genes (Osx, Runx2, CAP, CEMP and ALP) under the stimulation of CTGF(50ng/ml), Si-Cx43 and LiCl(5mM). *, P<0.05.
