# Supplementary material for: Connective tissue growth factor promotes cementogenesis and cementum repair via Cx43/β-catenin axis
Source: Stem Cell Res Ther. 2022 Sep 6;13:460. doi: 10.1186/s13287-022-03149-8 (PMC9450312; doi:10.1186/s13287-022-03149-8)
Supplement: Supplementary file 6 — Additional file 6. Sequences of primer pairs of housekeeping and mineralization-related genes in hPDLSCs for qPCR. [file 13287_2022_3149_MOESM6_ESM.docx]

**Supplemental table1.** Sequences of primer pairs of housekeeping and CCN, connexin and pannexin family genes in hPDLCs for qPCR

| Gene | Accession number | Primer pairs (5’-3’) |
| --- | --- | --- |
| GAPDH  ALP  COL1  RUNX2  CAP  SP7 (Osterix)  CEMP1 | NM_001289745.3  NM_000478.6  NM_000088.4  NM_001015051.4  NM_014241.4  NM_001300837.2  NM_001048212.3 | Forward: GACAGTCAGCCGCATCTTCT  Reverse: GCGCCCAA TACGACCAAA TC  Forward: CTATCCTGGCTCCGTGCTCC  Reverse: GTTAACTGATGTTCCAATCCTGCG  Forward: CCCCGAGGCTCTGAAGGT  Reverse: CACCAGCAATACCAGGAGCA  Forward:TCGCCTCACAAACAACCACA  Reverse: TCACCAGCAACTCCAACAGG  Forward: CCTGGCTCACCTTCTACGAC  Reverse: CCTCAAGCAAGGCAAATGTC  Forward: CTCCTGCGACTGCCCTA  Reverse: GCGAAGCCTTGCCATACA  Forward:TGAGATCAACCCCTTGTGCC  Reverse: GGCTCTGCCACTGTTCTCTT |
